# Event and Comment

**Published:** 1906-03-15

**Authors:** 


THE
### DENTAL REGISTER.

Vol. LX. March 15, 1906. No. 3.

### EVENT AND COMMENT.

### Army and Navy Dental Bills.

The Senate bill, which was introduced by Senator Pettus, and which seeks to put the army dentists on a commissioned basis rather than a contract basis, was passed in the Senate February 5th, but reconsidered the next day and put back on the calendar for future consideration. In the mean time the editor of the *Items of Interest*, has been scrutinizing the bill and finds it unsatisfactory, for the reason that it actually degrades the present organizers of the army dental corps from the rank and pay of major to the rank and pay of captains, with no possibility of promotion until ten more years of service have expired, by which time at least one of the members shall have reached the age for retirement. A careful reading of the bill justifies Dr. Ottolengui's criticism, and we most cordially sympathize with his view that steps should be taken to change this if it is practicable. Of course we don't understand the peculiar features of legislative enactments; and there seems to be an unusual difficulty in getting any reasonable legislation at Washington in connection with the army and navy affairs, because of the contemptible insolence that has grown up in army circles because of the pride of rank. It seems to us that unless the people of this country take hold of this matter and give army officers to understand once and for all that they are the servants of the people and not their masters, we shall soon have as deplorable a condition of affairs in this democratic land as now exists in Germany, where it is high treason for a civilian to attempt to assert his rights when an army man is concerned. The dental profession has wasted enough time over this affair, and it seems to us that it is high time we should get together and use all the power we possess to secure a just and proper enactment on the subject of both, the army and navy bills. Let us get these bills into proper form and have men of strength go down to Washington and present the bills with ample reasons for every detail, which it would seem there ought to be no great difficulty in doing. We want the dental corps in the army made up of our most thoroughly equipped young men, and in order to attract such men to the service, conditions must be made more acceptable than they now are, and the best possible means must be organized for determining who these men are. The hearing before the committee on the army bill should make it perfectly plain to everyone that the army and navy will never attract the best educated men unless they are given a reasonably dignified standing, and it will not need any very great argument to convince any intelligent statesman that the best possible professional talent should be enlisted to secure the entrance of only the highest type of professional men into this service. We therefore insist that there should be at least one dentist of known professional qualifications at the head of each division of the service, and that he should have a proper title and adequate compensation for his service. We could give good reasons for having the highest order of skill in the army and navy dental surgeons, but we haven't space, and besides, a great deal has been said, and more is being developed daily by the present corps of contract surgeons, which is doing its best under unreasonable handicaps, for the enlisted soldiers. If it is true that there is still time to change these bills, by all means let our committees get busy at once and if need be send to Congress our ultimatum.

### An Amendment to the Army Dental Bill.

To meet the criticism of the editor of the *Items of Interest*, and blaze the way for both Interstate and International reciprocity, we would suggest the following amendments to the present bills before Congress. First, that sections 2 and 4 of the army bill be amended so that, instead of making the three present members of the contract corps, who have been charged with the examination of candidates for the army service, captains, that one of them shall be appointed with the rank of major; and secondly, that he and an officer of similar rank in the navy corps, shall constitute the government members of a national board of dental examiners, which shall be further constituted by the appointment of three civilian dentists, one from the National Dental Association, one from the National Board of Dental Examiners, and one from the National Association of Dental Faculties. This commission should hold at least one session each year for the examination of graduates of standard colleges only, who have been at least two years in private and ethical practice. All graduates who shall pass this examination should receive a certificate, which shall exempt from the professional examination any who may desire to enter the army or navy service. Such a certificate may also be made the basis of reciprocity between the States of the United States, and would be of the greatest value to any dentist who might wish to obtain registration for practice in a foreign country, as it would have government sanction and would officially certify a definite standard of professional attainment. It would help us to go to foreign countries, where educational standards are made by the government, with an official standard that no country would be disposed to question. Reflexly it would raise the standard of professional education at home with a celerity and definiteness that none of the present methods will accomplish except after a long time. It seems to us that here is a grand opportunity to put our professional relations on an official basis; and to settle for all time the question of comity in our interstate and international relations. That there is great need that something shall be done to definitely specify our recognized standard of dental qualification abroad one need only to refer to the February *Dental Cosmos*, page 230, and read the article on "The D. D. S. Abroad," to learn that hundreds of American practitoners of good repute are about to have all desirable permission to persue their professional work in Germany taken from them, under the pretense that they have not earned their titles in recognizable institutions of learning. Here we have a chance to meet this opposition in a perfectly legitimate manner, and if we neglect to do so, we certainly cannot blame those who may for their own protection be constrained to discredit our educational system and our profession.

**Working for the Profession.**

We have just finished reading the fifty-page report of the hearing on the army dental bill before the United States Senate Committee, and while we see many points that seemed to be unnecessarily developed and other important ones that were not made as clear as they should have been, yet it seemed to us that the committee appreciated the effort made in its true light, and was convinced that the profession as a whole was sincerely backing the proposition. One of the very important features of this hearing was the large part taken in it by Dr. Wms. Donnally, and the complete understanding of every feature which he seemed to have at his tongues end when asked for. Whether Dr. Donnally ever sees the fruits of his labors or not, the untiring energy and tenacious manner with which he has pursued this cause ought to elicit the highest commendation for him in all future professional history. He has literally given his life to this work, and if the matter fails, the charge can never be laid at his door that he was unfaithful. If the profession had backed him up with one-half the energy that he has displayed, it is not at all unlikely that we should have had the desired legislation long ago. It is only another sample of how individuals in our profession have honored and advanced our profession at so great cost to themselves. We ought to appreciate our professional heroes more than we do.

**The Color Problem.**

A recent writer in one of our periodicals makes the statement that "Porcelain is the only filling material in use that gives us a choice of color. It is true that we do not always secure a perfect match in color, but even a harmony of color is a very decided gain over former efforts in which the color problem never entered as a factor. I anticipate in the near future a technical solution of the color problem in porcelain fillings." There is no apparent unrest in the minds of the average practitioner over this question, as the large majority of the profession are still ignoring it altogether in their unwillingness to abandon old practices for the new. But to the close observer it is very evident that there is a considerable awakening to the value of oral cosmetics in the practices of the most progressive practitioners. One who reads closely the proceedings of dental societies will find that the conspicuous gold crown has had its day with refined people, and it will in the near future pass from view altogether; he will also note that the black amalgams are being supplanted by amalgams of lighter colors, and that the large anterior fillings of gold are being replaced with porcelain inlays. In fact all objectionable colors are being forced into the background or eliminated altogether. This is certainly a grand triumph to the credit of the porcelain fillings, and should it prove in time to be an unreliable filling material, it will have accomplished one great work—it has unmistakably pointed out the barbarity of taste displayed in former operations. We sincerely trust our quoted author's expectations may be fulfilled, and that right quickly too, but we are convinced that the attainments already made are sufficiently good to answer all reasonable demands until the time may come when we shall know more about these problems. Dr. Byram is now studying the problem from the optical and temperamental standpoint, and his present elucidations are rather complex and hazy; yet from what we know of the man, we believe he will sooner or later have some message for us that will let in the light of science, which shall make the problem as easy as mixing paints. The color problem is an important one, but it is secondary, for the reason that even bad matches of color are so much more satisfactory than the metallic filling, that we shall suffer little from the failure to produce exactly matched shades in all cases.

### A New Form of Swindle.

A dentist by the name of F. W. Herr was detected taking the Pennsylvania State Board examination in the name of another dentist by the name of Senior, with the intent to pass the examination and turn over the registration license, to the man whose name he temporarily assumed, when it had been issued. He was arrested and charged with conspiracy to defraud. Now it seems that we have another prohibitory law that doesn't prohibit. This was either an unusually audacious or absurdly foolish piece of business. Which ever it may be, it should receive prompt and serious punishment that it may not occur again. Where did these young men learn to attempt such unfair and hazardous proceedings? Was it lack of home training, or have they been reading the tales of "frenzied finance," or were they graduates of some college that was so lax in discipline as to foster such habits?

